# Comparison of network structures between autistic and non-autistic adults, and autism subgroups: A focus on demographic, psychological, and lifestyle factors

**DOI:** 10.1177/13623613231198544

**Published:** 2023-09-29

**Authors:** Tulsi A Radhoe, Joost A Agelink van Rentergem, Carolien Torenvliet, Annabeth P Groenman, Wikke J van der Putten, Hilde M Geurts

**Affiliations:** 1 University of Amsterdam, The Netherlands; 2 Leo Kannerhuis (Youz/Parnassia Groep), The Netherlands

**Keywords:** adults, autism, network analysis, quality of life, subgroups, subtypes

## Abstract

Differences in (autism) characteristics are often reported between autistic and non-autistic adults but also between autistic adults. We aimed to determine whether mean differences correspond to differences in network structure of these characteristics in (1) autistic and non-autistic adults and (2) two previously identified autism subgroups. A total of 16 network variables related to demographic and psychological characteristics were included. First, Gaussian Graphical Models (GGMs) were used for network estimation in 261 autistic adults and 384 non-autistic comparisons aged 30–85 years. Second, we repeated this step within two previously identified autism subgroups (*N*_1_ = 124, *N*_2_ = 130). Third, sex differences were explored in the networks of the autism subgroups. The networks of the autism and comparison groups differed on individual edges and visual inspection, although the Network Comparison Test (NCT) showed no overall differences. The networks of autism subgroups were similar based on visual inspection and statistical comparisons. Sex did not impact the subgroup networks differently. Networks were more similar than different, but observed edge differences could be informative for targeted support. Focusing on mean differences is not sufficient to determine which factors (and associations) are important for autistic people. Thus, network analysis provides a valuable tool beyond assessing mean differences for autistic adults.

**Lay Abstract:**

There are large differences in the level of demographic, psychological, and lifestyle characteristics between autistic and non-autistic adults but also among autistic people. Our goal was to test whether these differences correspond to differences in underlying relationships between these characteristics—also referred to as network structure—to determine which characteristics (and relationships between them) are important. We tested differences in network structure in (1) autistic and non-autistic adults and (2) two previously identified subgroups of autistic adults. We showed that comparing networks of autistic and non-autistic adults provides subtle differences, whereas networks of the autism subgroups were similar. There were also no sex differences in the networks of the autism subgroups. Thus, the previously observed differences in the level of characteristics did not correspond to differences across subgroups in how these characteristics relate to one another (i.e. network structure). Consequently, a focus on differences in characteristics is not sufficient to determine which characteristics (and relationships between them) are of importance. Hence, network analysis provides a valuable tool beyond looking at (sub)group level differences. These results could provide hints for clinical practice, to eventually determine whether psychological distress, cognitive failures, and reduced quality of life in autistic adults can be addressed by tailored support. However, it is important that these results are first replicated before we move toward intervention or support.

The heterogeneity within the autism spectrum is a widely known phenomenon: autism is characterized by differences in core characteristics between autistic and non-autistic people but also by large differences between autistic people (Happé et al., 2006; Masi et al., 2017). For example, autistic adults report higher sensory sensitivity (Ben-Sasson et al., 2019; Crane et al., 2009) and more social interaction difficulties than non-autistic adults (Ruzich et al., 2015). However, there are also large individual differences between autistic people (Masi et al., 2017). While differences in the level of autism characteristics are informative as a description, they do not inform us about the mechanisms that give rise to these differences, nor do they provide specific leads for support or interventions. In this study, we do not focus on the heterogeneity in autism per se, but zoom in on this added level of complexity. Therefore, we focus on the relationship among characteristics instead of the level of characteristics, between autistic adults and non-autistic comparisons, and between autistic people.

A useful tool to gain more insight in the underlying structure of these differences is provided by the network approach. With a network analysis, one can visualize a complex system by identification of its components (i.e. nodes) and the relationships between these components (i.e. edges or links between nodes) (Borsboom & Cramer, 2013). The nodes can represent different types of things (e.g. persons, autism characteristics, cities) and the edges can represent different types of relationships (e.g. (partial) correlations, distances). One could use this method to examine whether there is a direct association between sensory sensitivity and difficulties with social interaction, or whether such an association is indirect (e.g. sensory sensitivity is associated with stress, while stress is associated with social interaction difficulties). An advantage of this method is that it also allows exploration of relationships in data when you do not have a clear hypothesis on how variables are related (Borsboom et al., 2021). In the autism field, it is often unknown how different types of characteristics are related; hence, this method could provide new insights into where autistic people might encounter difficulties and where autistic people flourish.

Network analyses have already proven insightful in autism. For instance, one study used a network analysis to elucidate risk and success factors for subjective wellbeing in autistic adults (Deserno et al., 2017). The study showed that social satisfaction and contribution to society were highly important for the wellbeing of autistic adults. Other studies focused on autism traits networks (Deserno et al., 2018; Montazeri et al., 2022) or mapped a network of autism traits and co-occurring psychological difficulties in children, such as anxiety (Montazeri et al., 2019) and obsessive-compulsive disorder (Ruzzano et al., 2015). A different study compared centrality indices in networks of autistic and non-autistic children and indicated that depression symptoms were more central in networks of autistic children (Montazeri et al., 2020), although this study did not involve a statistical comparison of the network structures. To our knowledge, there have not been any studies that included a formal statistical comparison of the network structures of autistic and non-autistic individuals. To know whether network structures generalize across (diagnostic) groups or whether they differ, formal statistical comparisons are needed. Moreover, the autistic population is marked by heterogeneity. To determine what is best for whom, it could well be that we need to estimate a network for each specific individual. However, to move away from (diagnostic) group level (i.e. one measurement per person), toward individual level (i.e. many measurements per person) is a big leap that requires a vast amount of data. Thus, before we turn toward the individual level, it is useful to take an intermediate step by first focusing on homogeneous subgroups.

Many studies have already focused on subgroup identification in autistic adults (for a review, see Agelink van Rentergem et al., 2021), by, for example, determining autism subgroups based on sensory sensitivity variables (DeBoth & Reynolds, 2017). In our own work, we identified two subgroups of autistic adults (Radhoe et al., 2023): a “Feelings of High Grip” subgroup and a “Feelings of Low Grip” subgroup, that differed on several variables (e.g. sensory sensitivity, social and communication skills, sense of mastery). These variables were included because they are important to aging while being autistic as they are considered risk/and or protective factors for psychological distress, cognitive failures, and reduced quality of life (Bishop-Fitzpatrick et al., 2017; Dallman et al., 2021; Frank et al., 2018; Hamm & Yun, 2019; Khanna et al., 2014; Mason et al., 2018; Pisula et al., 2015; Syu & Lin, 2018; van Heijst et al., 2020). Moreover, they show mean differences between autistic and non-autistic adults (Radhoe et al., 2023). The “Feelings of Low Grip” subgroup had the most vulnerable profile on the included measures and scored less favorable on external, clinical outcomes: quality of life, psychological difficulties, and cognitive failures. This shows that subgroups of autistic adults show differences in the level of autism characteristics (i.e. mean differences) and other psychological factors. It is not yet known how the network approach and subgrouping approach which looks at mean differences could complement one another.

There are different ways in which mean differences could correspond to differences in network structure. It could be that the subgroups differ in the level of certain factors, while the relationship between these factors is comparable within each subgroup. However, it could also be that observed mean differences between subgroups go together with a difference in (causal) relationship between factors. In a given subgroup, subgroup A, there may be a direct relationship between sensory sensitivity and social interaction, that is, sensitivity to noises (and other sensory stimuli) leads to problems with having conversations in loud environments. In subgroup B, there may be an indirect relationship between sensory sensitivity and social interactions, that is, sensory sensitivity might lead to avoidance behavior toward social events, leading to more social interaction difficulties. In the latter subgroup, this would imply that sensory sensitivity and social interaction difficulties are conditionally independent given the avoidance behavior (Borsboom & Cramer, 2013). The network approach can be utilized to assess this type of differences between data-driven subgroups.

In this preregistered study (Aspredicted #49209), we aim to determine how these techniques complement each other by combining our subgrouping approach with the network approach. First, we test at the (diagnostic) group level whether there are differences in network structure between autistic adults and non-autistic comparisons. Second, we zoom in at the subgroup level by testing for differences in networks between the previously identified autism subgroups (Radhoe et al., 2023). Figure 1 shows an illustration of the relationship between statistical methods. Third, we exploratively assess the impact of biological sex on the networks of the autism subgroups as differences between autistic men and women in behavioral characteristics have often been reported (see, for example, Baron-Cohen et al., 2014; Werling & Geschwind, 2013). Specifically, we aim to determine whether there are (sub)group differences in (1) the overall network structure and/or (2) specific relationships between factors that are important to aging while being autistic (such as between sensory sensitivity and social interaction difficulties). This study will bring to light whether mean differences correspond to differences in network structure in autistic adults and non-autistic comparisons, potentially improving our understanding of risk and protective factors and targeted support.

**Figure 1.**
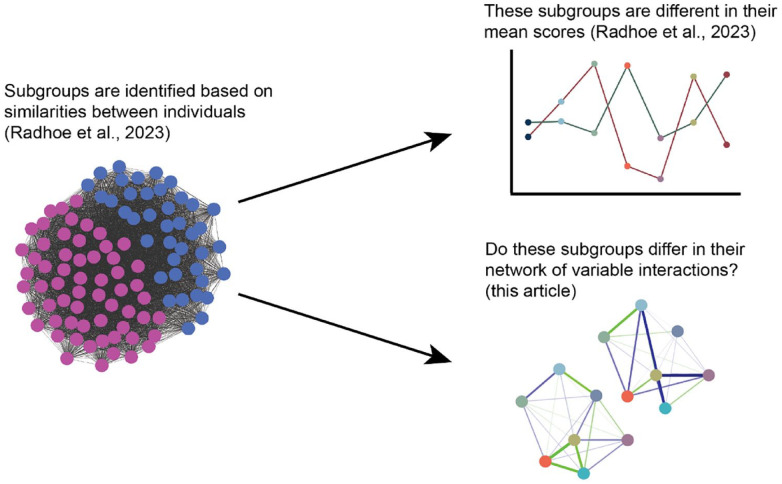
Illustration of the relationship of this article to previous work.

## Methods

### Participants

In total, 661 adults participated in this study: there were 261 adults in the autism group (AUT; 133 women, 127 men, 1 other) with a mean age of 51.2 years (SD = 12.7, range = 30–84) and 384 adults in the comparison group (COMP; 170 women, 214 men) with a mean age of 54.9 years (SD = 13.8, range = 30–85). We did not ask participants about race/ethnicity and socioeconomic status, but the majority of the participants was White and had a high educational attainment (please see our earlier work for a more detailed sample description (Radhoe et al., 2023)).

For both groups, we applied the following exclusion criteria: (1) present or past diagnosis of intellectual disability or an IQ-score below 70, (2) being younger than 30 years, and (3) insufficient understanding of Dutch language required to complete the questionnaires. In the autism group, we only included participants with a clinical diagnosis of an autism spectrum disorder (ASD) according to the *Diagnostic and Statistical Manual of Mental Disorders* (4th ed., DSM-IV or 5th ed., DSM-5; American Psychiatric Association, 2000, 2013). To optimize the likelihood of observing differences between the autism and comparison group, we applied the following addition exclusion criteria to the comparison group: (1) a present or past ASD diagnosis or a total score higher than 32 on the Autism Spectrum Quotient (Baron-Cohen et al., 2001), (2) an ASD diagnosis in close family members (i.e. parent(s), child(ren), sibling(s)), (3) a history of more than one psychotic episode, (4) a present or past diagnosis of attention-deficit/hyperactivity disorder (AD(H)D) or a total score of six or higher on the Dutch version of the ADHD DSM-IV Rating Scale (Kooij et al., 2005), and (5) AD(H)D in close family members.

Participants in the autism group were recruited through mental health institutions in the Netherlands and advertisements placed on social media and client organization and autism advocacy websites. Participants in the comparison group were recruited through advertisements on social media and via the social network of the researchers, research assistants, and students collaborating on this study in the period between 2018 and 2020.

### Autism subgroups

In our earlier work, we focused on subgroup identification within the autism group (Radhoe et al., 2023). We identified two subgroups of autistic adults: a “Feelings of High Grip” subgroup^1^ (*N* = 124, mean age = 50.6, SD = 13.8, range = 30–81, 47% women) and a “Feelings of Low Grip” subgroup (*N* = 130, mean age = 51.3, SD = 11.6, range = 30–84, 55% women). In this study, we proceeded with both (1) the overall diagnostic groups (described above) and (2) the identified autism subgroups.

### Measures

We included 17 variables in the network analysis. The first 11 variables were previously included in the identification of the subgroups. The last variable, biological sex, is included only in a separate analysis. The measures had sufficient psychometric qualities based on the general population and autistic population. Supplementary Material S1 includes detailed information on the psychometric and distributional properties of the included measures:

Autism characteristics were measured using two instruments. First, the Autism Spectrum Quotient (AQ) (Baron-Cohen et al., 2001) was used by including of subscale scores for “Social interaction” (40 items, range = 0–40) and “Attention to detail” (10 items, range = 0–10) (Hoekstra et al., 2008). A higher score reflects more autism characteristics. Second, the Sensory Sensitivity Questionnaire (SSQ) (Lever & Geurts, 2013; Minshew & Hobson, 2008) was used to measure sensory sensitivity. A total score was included (13 items, range = 0–13), where a higher score was indicative of a higher sensory sensitivity level.
Educational level was measured by asking participants about the highest education degree they obtained. We used the Dutch Verhage scale to classify the educational level (Verhage, 1964), consisting of seven categories (i.e. 1 = less than 6 years of primary education, 7 = university degree).
Sense of Mastery (was measured by the Pearlin Mastery Scale (Pearlin et al., 1981)). Mastery is the extent to which we consider ourselves as being in control of our lives. We included the sum score (7 items, range = 7–35) where a higher score is indicative of a higher sense of mastery.
Worries/Fears were measured by a combination of the Worry Scale (Wisocki et al., 1986) and Fear Questionnaire (Marks & Mathews, 1979). The total score was included (15 items, range = 15–75). A higher score indicates more worries/fears.
Physical activity was assessed using the International Physical Activity Questionnaire (IPAQ) (Craig et al., 2003). We included the total amount of minutes during which a participant was physically active during the past week (11 items, range 0 to the maximum number of minutes per week, i.e. 10,080). A higher score reflects more physical activity.
Negative life events were measured using the List of Threatening Experiences (Brugha et al., 1985). Based on 12 different life events, a total score was included (12 items, range 0 (no negative life events) to 12 (many negative life events)).
Emotional support was assessed using the Close Persons Questionnaire (Stansfeld & Marmot, 1992). For emotional support, a total score was included (12 items, range = 12–60). A higher score indicates a higher level of emotional support people felt that they received.
Positive affect (PA) and negative affect (NA) were measured using the Positive and Negative Affect Schedule (PANAS) (Watson et al., 1988). We included scores for PA (10 items, range = 10–50) and NA (10 items, range = 10–50). A higher score indicated more positive (or negative) affect.
Cognitive failures were measured using the Cognitive Failures Questionnaire (Broadbent et al., 1982). A total score was included (25 items, range = 0–100). A higher score indicates more experienced cognitive failures.
Quality of life was measured using the World Health Organization Quality of Life Questionnaire-BREF (WHOQoL-BREF) (Harper, 1998). We included the first question referring to one’s overall quality of life perception (one item, range = 1–5), where a higher score is indicative of a higher quality of life.
Psychological difficulties were assessed using the Symptom Checklist-90 Revised (SCL-90-R) (Derogatis, 1977). A total score was included (90 items, range = 90–450), where a higher score is representative of more psychological difficulties. The SCL-90-R screens for a variety of psychological difficulties related to somatization, obsessive compulsion, depression, anxiety, interpersonal sensitivity, hostility, phobic anxiety, paranoid ideation, and psychoticism.
Number of physical illnesses was measured using the Health Interview Questionnaire (Central Bureau of Statistics [CBS], 1989). A total score was included (21 items, range = 0–21). Like most previous measures, a higher score reflects more reported physical illnesses.
Age was measured by asking participants about their chronological age.
Biological sex was measured by asking participants about their biological sex. Response options were “male,” “female,” and “other.” Therefore, we included this variable as a categorical variable in the network analysis.

### Procedure

We refer to the published protocol for the detailed procedure (Geurts et al., 2021), but below the procedure is described briefly. After receiving written informed consent, participants filled out a set of questionnaires (online or on paper, dependent on the participant’s preference). If participants met the inclusion criteria, they filled out a second set of questionnaires. They received €7.50 for completing the questionnaires. This study was approved by the local ethics review board of the department of Psychology of the University of Amsterdam (2018-BC-9285).

### Statistical analyses

All analyses were conducted in RStudio version 1.3.1073 (RStudio Team, 2020). We preregistered the analysis plans at AsPredicted.org (AsPredicted #49209, https://aspredicted.org/ISU_KHS). The analysis can be described in four parts: (1) missing data, (2) network methods, (3) network comparisons, and (4) power analysis. We followed the reporting standards for psychological network analyses (Burger et al., 2022).

#### Missing data

We based our dealing with missing data on the simulation studies described below (for more information, see also Supplementary Material S3). Within a specific questionnaire, we considered 10% of missing data per respondent appropriate for imputation at the item level (Bennett, 2001). The manner of imputation depended on the measurement instrument. For autism characteristics, mastery, worries, emotional support, PA and NA, psychological difficulties, and cognitive failures, we recoded a maximum of 10% of missing values to the median of the participant’s other responses on that specific measurement instrument. For negative life events, number of physical illnesses, and physical activity, we recoded a maximum of 10% of missing values to zero, which implies the absence of a negative life event, physical illness, or physical activity. We did not impute any missing values on education, quality of life, biological sex, and age. After our item-level imputation was completed, we removed participants who still had missing values on more than one network variable (out of 16 variables in total) from all analyses. Thus, information was available from each respondent on at least 15 network variables.

As the NCT does not handle missing data, we excluded participants with any missing observations before we proceeded to the statistical comparison of the networks.

#### Network estimation methods

A network consists of nodes (i.e. variables) connected by edges (statistical relationships between nodes) (Hevey, 2018). In this study, the edges represent partial correlations that indicate the strength of a relationship between two nodes after controlling for the effects of all other associations in the network (Epskamp & Fried, 2018). We used two different models to estimate the networks depending on the specific research question and the type of data. First, we used GGMs when estimating the networks using continuous data (*bootnet* package v1.5; Epskamp et al., 2018). Second, Mixed Graphical Models (MGMs) were used when we estimated the networks using both continuous and categorical data (*mgm* package v1.2-12; Haslbeck & Waldorp, 2020). The *qgraph* package was used for network visualization (v1.9.2; Epskamp et al., 2012).

A common problem in network estimation is the existence of spurious edges or false positives (Epskamp & Fried, 2018). This implies that you obtain small partial correlation coefficients, even when two variables are conditionally independent (i.e. two variables are independent after controlling for the effects of all other variables in the network). These spurious edges complicate the interpretation of the network and may lead to failures to replicate estimated networks (Epskamp & Fried, 2018). To limit the number of spurious edges, we applied the *least absolute shrinkage and selection operator* (lasso) as a method of regularization. The lasso only retains the most robust edges by shrinking all estimates, causing some estimates (such as the smaller, spurious edges) to become exactly zero (Deserno et al., 2017). The lasso utilizes a tuning parameter, λ, to determine the level of sparsity. It is important to select the appropriate value of λ, such that the number of spurious edges is minimized while the number of true edges is maximized. We used the Extended Bayesian Information Criterion (EBIC) to determine the value of λ (for more detail, see Epskamp & Fried, 2018). After estimating the network structure, the accuracy of the edge weights was examined by estimating a 95% confidence interval (CI) around the edge weights (using non-parametric bootstrapping with 2500 bootstrap samples; *bootnet* package v1.5; Epskamp et al., 2018). This procedure allows assessment of the variability in the edge weights and to compare edges with each other. The wider the bootstrapped CIs, the more difficult it becomes to interpret the strength of an edge.

Two centrality indices were calculated in order to interpret the networks (Costantini et al., 2015; Opsahl et al., 2010; Robinaugh et al., 2016): (1) strength (i.e. how well a node directly connects to other nodes) and (2) expected influence (i.e. the influence of a node on its immediate neighbors). Node strength quantifies the sum of the weights of the connections to other nodes in absolute value (Costantini et al., 2015). A node with relatively high strength may influence many other nodes, without requiring the mediating role of other nodes in the network. The expected influence considers both positive and negative edges (rather than focusing on the absolute sum of edge weights as is the case with node strength). Hereby it does not only consider the position within a network but also the nature of its connections to other nodes in the network. Consequently, expected influence may be better at identification of nodes that are highly influential in networks that also include negative connections (Robinaugh et al., 2016). In contrast to what was preregistered, we did not report closeness and betweenness as centrality indices as recent studies have indicated that their use should be discouraged (Bringmann et al., 2019). The stability of the centrality indices was assessed using the correlation stability (CS) coefficient (*bootnet* package version 1.5; Epskamp et al., 2018). The CS-coefficient represents the maximum proportion of participants than can be dropped from the data set, such that (with 95% probability) the correlation between the original centrality indices and those based on the subset is 0.7 or higher. Based on a simulation study, CS-coefficients should not be below 0.25 in order to be considered stable indices (Epskamp et al., 2018).

#### Network comparisons

Three different comparisons were performed based on the research questions. First, to determine whether the network structure generalized across diagnostic groups, a GGM was estimated separately in the full autism and comparison groups. Sixteen variables were included in this analysis (i.e. the variables mentioned under “measures,” except for biological sex). After estimating the networks, different methods may be used to compare the networks, such as a NCT (van Borkulo et al., 2022) or a moderation analysis that can be used to compare more than two groups (Haslbeck, 2022). In this study, we aimed to compare two groups and therefore applied the most commonly used method for network comparison; the NCT was used to assess differences across diagnostic groups (*NetworkComparisonTest* package v2.2.1; van Borkulo et al., 2022). Two outcomes are of importance for this test: (1) difference in network structure (i.e. are the distributions of edge weights comparable across the two networks) and (2) global strength (i.e. is the overall level of connectivity equal across the two networks). The first outcome relates to the structure of the network as a whole, and tests whether this structure is completely identical across groups (van Borkulo, 2017). The second outcome demonstrates whether the overall level of connectivity is equal across groups, even though the networks may still differ in network structure. Consequently, both outcomes are used to test distinct hypotheses. Moreover, differences in individual edges between the networks were tested using the NCT. We also explored the results visually.

Second, to gain further insight at the subgroup level, GGMs were estimated in the previously identified autism subgroups using the aforementioned sixteen variables as input. Again, an NCT was used to test for differences between the autism subgroups.

Third, to assess whether biological sex (a categorical variable) had a differential impact on the networks of the subgroups, an MGM was used. Seventeen variables were included, that is, all variables mentioned under “measures.” For this analysis, we did not separately consider centrality measures again. Differences between the networks were assessed using an NCT.

#### Power analysis

We performed four simulation studies (*parSim* package v0.1.4.; Epskamp, 2020) to assess whether we had sufficient power to estimate a network with 16 nodes (i.e. the before-mentioned variables excluding sex) for each subgroup and to compare the networks. In these simulations, we varied the sample size, percentage of missing data, and two parameter values that can be used to reduce the number of edges (for a detailed description of the simulations, please refer to Supplementary Material S2). The simulations indicated that we were able to estimate and compare networks for 120 participants per subgroup with a maximum of 10% missing data, a sensitivity of 0.50, a specificity of 0.90, and a false discovery rate of 4% (with specific parameter values for reducing the number of edges, namely, alpha = 0.25, gamma = 0).

### Community involvement

We worked together with a group of older/autistic adults (also referred to as the think tank) for this specific study and our overall study on aging in autism (Geurts et al., 2021). We met with the think tank at least three times a year (either online or in person) to discuss the recruitment strategies, information for participants, the interpretation of study results, and other study-related matters. For this specific study, the think tank gave their interpretation of important associations in the networks and differences between the networks during an in person meeting. The members were paid for their contributions.

## Results

After dealing with missing data (i.e. removing participants who still had missing values on more than one network variable), the autism network included 258 adults and the comparison network included 287 adults. The network of the HighGr subgroup included 123 autistic adults, and for the LowGr subgroup, 128 autistic adults were included. Specific information on missing data is provided in Supplementary Material S3. The main findings are described below, but please refer to the Supplementary Material for all plots of centrality indices, results of the non-parametric bootstrap analyses, and results of the NCTs (i.e. Supplementary Material S4–S10).

### Psychological difficulties are central for autism group

In the autism group, the total amount of psychological difficulties had the highest values on node strength and expected influence measures (CS > 0.25). Education and age had low values on the centrality indices suggesting that differences in these demographics cannot explain associations between variables.

**Figure 2.**
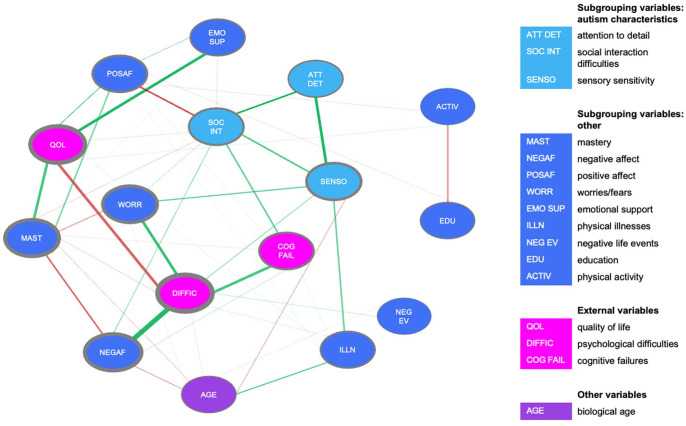
Estimated network based on data from the autism group. The color of the edge indicates the direction of the association (green for positive associations; red for negative associations). The thickness of the edge indicates the strength of the association: the thicker the edge, the stronger the association. The gray borders around the nodes indicate the strength of the node; the thicker the border, the higher node strength.

The autism group network is depicted in Figure 2. Psychological difficulties were associated with NA and worrying. Quality of life was not directly related to NA, but was positively related to other variables that could be considered aspects of wellbeing, like feelings of mastery and PA. Sensory sensitivity was associated with attention to detail and difficulties with social interactions—these three variables are all autism characteristics. Four of these associations were reliably the strongest in the network (i.e. their bootstrapped CIs did not overlap with those of most other edges): psychological difficulties with NA, attention to detail with sensory sensitivity, quality of life with mastery, and psychological difficulties with worrying.

### Differences at the edge level between autism and comparison groups

In the comparison group, the total amount of psychological difficulties also had the highest value on the centrality indices (CS > 0.25). The number of negative life events and education scored lowest on all centrality parameters, which indicates that these variables do not explain associations between the other variables.

**Figure 3.**
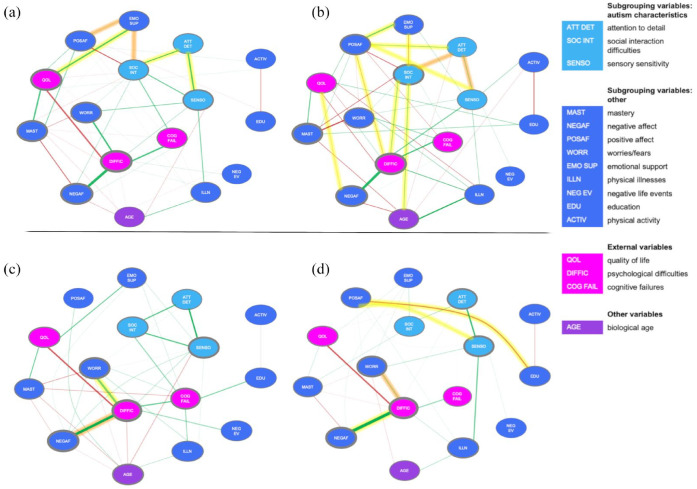
(a) Estimated network based on data from the autism group. (b) Estimated network based on data from the comparison group. (c) Estimated network based on data from the “Feelings of High Grip” subgroup. (d) Estimated network based on data from the “Feelings of Low Grip” subgroup. The highlighted edges indicate significant edge differences between the groups (i.e. autism vs comparison or HighGr vs LowGr): yellow indicates a higher value compared to the other group, and orange indicates a lower value compared to the other group. The color of the edge indicates the direction of the association (green for positive associations; red for negative associations). The thickness of the edge indicates the strength of the association: the thicker the edge, the stronger the association. The gray borders around the nodes indicate the strength of the node; the thicker the border, the higher node strength.

The comparison group network is displayed in Figure 3(b). Similar to the autism group, there was a positive association between psychological difficulties and NA. The number of physical illnesses was negatively associated with quality of life and positively with psychological difficulties and age. Thus, more self-reported physical illnesses were associated with lower quality of life, more self-reported psychological difficulties, and a higher age. PA was related with most psychological variables in the network: positively related to quality of life, emotional support and mastery, and negatively associated with worrying and psychological difficulties. Three of these associations were reliably the strongest in the network according to the non-parametric bootstrap analysis: psychological difficulties with NA, physical illnesses with age, and emotional support with PA.

Based on a visual inspection of the autism network (Figure 3(a)) and the comparison network (Figure 3(b)), it seemed that the comparison network was more connected, whereas the autism network included stronger connections.

However, a statistical comparison (*N*_autism_ = 233, *N*_comparison_ = 254) showed that there were no significant differences in global strength (*p* = 0.36) and in network structure (*p* = 0.09). This implies that the autism and comparison groups did not differ in (1) the overall level of connectivity and (2) the manner in which the edge weights were distributed across the networks (i.e. network structure). Although there were no overall differences between the networks, the NCT indicated several differences in individual edges (see highlighted edges in Figure 3(a) and (b)). As expected, in the autism group, the autism characteristic variables (i.e. attention to detail, social interaction difficulties, and sensory sensitivity) were more strongly connected compared to the comparison group. In the comparison group, the variables related to an autism diagnosis (i.e. attention to detail, social interaction difficulties, and sensory sensitivity) showed more connections to other network variables compared to the autism group. Also, there were stronger associations between PA and (1) emotional support, (2) psychological difficulties, (3) sensory sensitivity, and (4) attention to detail compared to the autism group. Thus, although there are no structural differences between the autism and comparison group, there were differences at the edge level.

### From a network perspective autism subgroups do not structurally differ

For both autism subgroups (i.e. the “Feelings of High Grip” (HighGr) and “Feelings of Low Grip” (LowGr) subgroups), the total amount of psychological difficulties had the highest value on measures of node strength, as was the case in the total autism group. This indicates that psychological difficulties were central in the subgroup networks and in the total autism group. Physical activity and the number of negative life events had the lowest values on the centrality measures (CS > 0.25).

In both subgroups, there were strong positive associations between (1) psychological difficulties and NA and (2) attention to detail and sensory sensitivity. In the HighGr subgroup, there was an additional strong positive association between psychological difficulties and worrying.

A visual examination indicates that there were more and stronger connections in the HighGr subgroup as compared with the LowGr subgroup. A statistical comparison (*N*_HighGr_ = 110, *N*_LowGr_ = 117) showed that the networks did not differ in global strength (*p* = 0.66). This indicates that the overall level of connectivity was equal between the subgroups. No differences were found in network structure (*p* = 0.58), indicating that the distribution of edge weights was similar across subgroups. Although there were no overall differences between the networks, a few individual edges differed between the subgroups according to the NCT. In the HighGr subgroup, there was a stronger positive association between psychological difficulties and worrying. In the LowGr subgroup, there was a stronger association between psychological difficulties and NA. Moreover, there was a stronger association in the LowGr subgroup between PA and (1) education and (2) sensory sensitivity. Thus, in the LowGr subgroup, PA relates to more variables compared with in the HighGr subgroup. However, overall, as aforementioned, both subgroups do not fundamentally differ from a network perspective.

### Biological sex does not affect the autism subgroup networks differently

**Figure 4.**
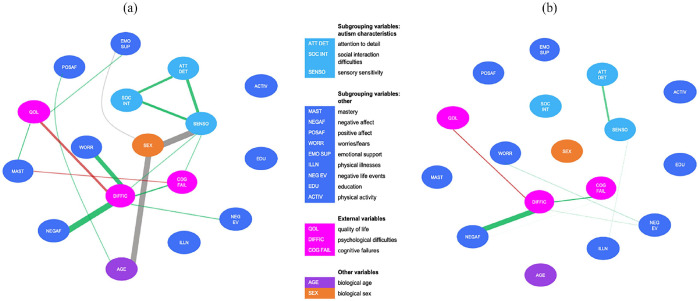
(a) Estimated network (including biological sex) based on data from the “Feelings of High Grip” subgroup. (b) Estimated network (including biological sex) based on data from the “Feelings of Low Grip” subgroup. The color of the edge indicates the direction of the association (gray indicates associations with sex, a categorical variable; green indicates positive associations; red indicates negative associations). The thickness of the edge indicates the strength of the association: the thicker the edge, the stronger the association.

In the HighGr subgroup, adding biological sex (i.e. male vs female) to the network resulted in three additional significant associations. The network is displayed in Figure 4(a). First, sex was associated with age, indicating that women were younger in this subgroup than men. Second, there was an association between sex and sensory sensitivity, which indicates that women reported higher sensory sensitivity compared with men in this subgroup. After adding sex to the network, there was no longer a significant association between age and sensory sensitivity. Thus, sex mediated this association. Third, sex was related to emotional support, indicating that women experience more emotional support than men. Moreover, after adding sex to the network, the association between sensory sensitivity and emotional support became absent, demonstrating that sex mediated this relationship. In the LowGr subgroup, adding biological sex to the network did not result in any additional associations. The network is depicted in Figure 4(b).

Thus, while adding sex resulted in several changes in the HighGr subgroup, it did not influence the network of the LowGr subgroup. Nonetheless, a statistical comparison did not indicate a significant difference between the two networks with respect to global strength (*p* = 0.15) and network structure (*p* = 0.27).

## Discussion

In this study, we focused on network structures to determine whether mean differences between (sub)groups correspond to differences in network structures. When comparing the networks based on data from self-report questionnaires, we found that (1) networks of autistic adults and non-autistic adults did not differ in overall structure, but showed differences in individual edges and (2) the autism subgroups that showed mean differences in various aspects, hardly differed in their network structure. Moreover, adding biological sex did not impact the networks of the autism subgroups differently. We did observe some subtle differences between the identified subgroups that provide hints for both clinical practice and future research. The LowGr subgroup reported more psychological difficulties and cognitive failures and a lower quality of life (Radhoe et al., 2023). The network of this subgroup showed more associations with PA (i.e. with sensory sensitivity and education). Even though we did not find differences in overall network structure, it would be premature to conclude that the networks of the autism subgroups can be merged into a single network.

When comparing the networks of the overall autism and comparison groups, we conclude that these networks differ as (1) the networks seem to differ in connectivity based on visual inspection and (2) while the NCT indicated no overall differences, there were differences in individual edges. Specifically, in the comparison group, there were both more and stronger connections as compared to the autism group. When comparing the networks of the autism subgroups, we conclude that the networks do not differ as (1) the NCT indicated no significant differences, (2) the networks seem similar based on visual inspection, and (3) there were hardly any differences in individual edges. Thus, networks between an autistic and a non-autistic group show some subtle differences while this is not the case for subgroups within the autism group.

In contrast to what we expected, some of the factors on which the autism subgroups showed large mean differences in our previous study (i.e. social interaction difficulties, mastery, and emotional support) were not the factors that showed differences in connectivity in this study. This does not imply that these variables are not important for autistic adults. In fact, the opposite may be true: while the connectivity of mastery does not differ between the networks, this factor is connected to many other variables in both subgroups. This stresses the importance of mastery for autistic adults in general (i.e. not dependent on subgroup membership). This has also been indicated in earlier research in autistic adults as mastery was found to be an important node connecting depressive symptoms with autism characteristics (van Heijst et al., 2020).

There were also some variables that showed large mean differences and showed differences in connectivity in the networks (i.e. worrying, PA and NA, and sensory sensitivity). Thus, while we initially thought that factors with large mean differences would correspond to either (1) differences in connectivity between the networks (because these factors would be most important) or (2) no differences in connectivity (because the variation in this factor would already be explained by the subgrouping solution), this study indicates that these two aspects are unrelated. Thus, based on mean differences, one cannot make any claims about the relationships of these factors, emphasizing the added value of the network approach.

This study has several strengths. First, it is a novel approach to test whether the findings of our subgrouping study on autism can be validated when followed by a network comparison. Rather than limiting our conclusions to mean differences between (sub)groups, we gained additional insight by considering the associations between factors. Second, we included a large sample of autistic adults across a wide age span from 30 to 85 years. These adults were recruited from the community and from mental health institutions to obtain an accurate representation of the diverse population of autistic adults (without an intellectual disability) in the Netherlands. Third, we preregistered our analysis plan (AsPredicted #49209, https://aspredicted.org/ISU_KHS), which is especially important given the exploratory nature of this study. Fourth, we preceded our network analysis by detailed simulations to determine whether we had sufficient statistical power to perform the desired analyses. Fifth, we discussed our findings with our think tank of older/autistic adults to help us interpret our results.

However, there are also several caveats that need to be acknowledged. First, it proved difficult to interpret subgroup differences, as (1) we did not find any overall differences according to the NCT and (2) adding biological sex to the subgroup networks had a complex effect. Specifically, biological sex visually seemed to affect the network structure, which shows that adding one additional variable (e.g. biological sex) could impact the associations in the networks. This indicates that one should only make claims based on the variables that are included in the network analysis. Nonetheless, we did find differences in individual edges between the subgroups that could be interesting for clinical practice. Second, the interpretation of psychological networks in general can be challenging, as it is difficult to decide what associations to focus on and to extract their meaning correctly (Bringmann & Eronen, 2018). Hence, we considered it essential to discuss our findings with our think tank of older/autistic adults. Third, there was a non-trivial reduction in sample size in the comparison group due to missing data that could not be imputed (i.e. from 384 to 287). This was mostly due to participant’s having missing data on (1) all questionnaires (except for participant’s age and sex) or (2) the entire second questionnaire booklet that included over half of the measures. Consequently, as missing data were not disproportionately high on a specific questionnaire, this was not considered an issue for the network analysis. On a related note, the imputation strategies that were adopted to deal with missing data differed between measurement instruments. For most instruments, median imputation was used. However, for negative life events, physical activity, and physical illnesses, the content of the questionnaires did not seem appropriate for (median) imputation based on the participant’s responses on the other items on that specific questionnaire. For example, imputing a missing value on “Did you ever have cancer?” based on the response on “Did you ever have high blood pressure?” did not seem correct. For these questionnaires, missing values were recoded to zero, implying the absence of a negative life event, physical activity, or physical illness. While this imputation strategy seemed most correct given the content of the questionnaires, recoding these missing values to zero may have led to under-reporting on these questionnaires.

Fourth, in all estimated networks (i.e. autism, comparison, HighGr, LowGr), we found a strong positive association between NA and psychological difficulties. The more negative emotions (e.g. anger, disgust) one has, the more psychological difficulties (e.g. sleep difficulties) or vice versa. Thus, this association is not specific to autism (or to an autism subgroup) but applies to both autistic and non-autistic people. The high correlation between these factors may induce questions regarding the distinctiveness of these concepts. Although these concepts are related, there is a clear difference. Psychological difficulties, measured with the SCL-90-R (Derogatis, 1977), reflects *current* distress (i.e. during the past week), so this is sensitive to fluctuations in mood. In contrast, NA, measured with the PANAS (Watson et al., 1988), reflects a *general* dimension of aversive mood states, representing a trait-like stability. As an example, one could imagine that losing your job could cause sleep problems and depressive feelings for a while, although this does not mean that you experience these emotions this intensively in general. Thus, one’s general state may not be identical to one’s current state of distress. Therefore, separate measures are justified.

Some might argue that additional exclusion criteria applied to the comparison group may affect study findings. To optimize the chance of discovering true differences between the autism and comparison group, those with undiagnosed autism needed to be excluded from the comparison group. Therefore, additional criteria were applied to the comparison group (e.g. presence of AD(H)D). The stricter exclusion criteria for the comparison group as opposed to the autism group may have resulted in differences beyond being autistic or non-autistic. A known problem in the autism field is the frequent under-recognition and subsequent underdiagnosis of autism in adulthood (O’Nions et al., 2023). In this study, we have tried to eliminate undiagnosed autism by extra exclusion criteria for the comparison group. However, in the attempt to correct for occurrences of undiagnosed autism in the comparison group, we may have introduced unintended differences between the autism and comparison group, for example, driven by the presence of AD(H)D. Nonetheless, we consider the impact of these differences smaller and less extreme than they would have been without any correction (which would have led to more autistic adults being included in the comparison group).

To explore whether biological sex had a differential impact on the networks of the subgroups, an MGM model was estimated. This analysis was conducted after using a GGM to estimate the subgroup networks without biological sex included. This may lead to some considering the GGM model redundant (i.e. the model without biological sex). Both network models were included in the manuscript for varying reasons. First, the analyses regarding the GGM and MGM were both preregistered. Furthermore, as the main research question was not aimed at the influence of sex, it was more suitable to address this question in an additional exploratory analysis using the MGM. Finally, the sample size justification was based on a GGM with 16 nodes (see Supplementary Material S2). Nonetheless, the more elaborate model with more variables supersedes the simpler model.

Some of the edge differences between the autism subgroups could be informative for clinical practice. For example, if an autistic adult from the LowGr subgroup seeks help in clinical practice, it may be wise to be aware of vulnerabilities in PA and NA, as these variables are strongly related to other important factors (e.g. worrying, psychological difficulties, and sensory sensitivity). The focus on identifying factors that may have a role in the development of (psychological) problems is also in line with the clinical guideline for autistic adults (National Institute for Health and Care Excellence, 2012). However, we do not yet know whether intervening on these factors indeed has the desired impact on the other factors. When the current findings are replicated, it could be fruitful to test whether targeting PA/NA indeed has an impact on the experience of psychological difficulties.

Moreover, future research focusing on qualitative data may be valuable for clinical practice in the long run. Based on previous research and the findings of this study, we have established *that* the autism subgroups differ, but it is still unknown *what gives rise* to these differences. Future studies aimed at qualitative data may produce valuable insights into the causal mechanisms that underlie the differences between these subgroups. For example, autistic adults in the HighGr subgroup may have a different (i.e. more beneficial) way of dealing with difficulties in daily life than autistic adults in the LowGr subgroup. This type of information may eventually be informative for developing interventions in (clinical) practice.

In conclusion, we showed that network analysis provides a valuable tool beyond looking at mean differences when comparing (sub)groups. While we expected that there was some relationship between mean differences and corresponding network architecture, this study showed that these aspects are unrelated. Thus, to make claims about the importance of certain factors (and the associations between these factors) for autistic people, solely looking at mean differences is not sufficient. Future studies should first focus on replicating these results before moving to intervention, to eventually determine whether distress, cognitive failures, and reduced quality of life in autistic adults can be addressed by the provision of tailored support.

## Supplemental Material

sj-docx-1-aut-10.1177_13623613231198544 – Supplemental material for Comparison of network structures between autistic and non-autistic adults, and autism subgroups: A focus on demographic, psychological, and lifestyle factors Supplemental material, sj-docx-1-aut-10.1177_13623613231198544 for Comparison of network structures between autistic and non-autistic adults, and autism subgroups: A focus on demographic, psychological, and lifestyle factors by Tulsi A Radhoe, Joost A Agelink van Rentergem, Carolien Torenvliet, Annabeth P Groenman, Wikke J van der Putten and Hilde M Geurts in Autism

## Notes

1. The labels of the subgroups were suggested by our think tank of older/autistic adults and were based on the cluster variables on which the subgroups differed significantly (for more information, see Radhoe et al., 2023). It is important to be aware of the possible impact of using subgroup labels on the interpretation and conclusions drawn. Thus, the suggested labels should not be used outside the context of this study.
