# Supplementary material for: Comparison of network structures between autistic and non-autistic adults, and autism subgroups: A focus on demographic, psychological, and lifestyle factors
Source: Autism. 2023 Sep 29;28(5):1175–89. doi: 10.1177/13623613231198544 (PMC11067416; doi:10.1177/13623613231198544)
Supplement: sj-docx-1-aut-10.1177_13623613231198544 – Supplemental material for Comparison of network structures between autistic and non-autistic adults, and autism subgroups: A focus on demographic, psychological, and lifestyle factors [file sj-docx-1-aut-10.1177_13623613231198544.docx]

Supplementary materials for:

**Comparison of network structures between autistic and non-autistic adults, and autism subgroups: A focus on demographic, psychological and lifestyle factors**

**Table of content**

**S1) Measures: Psychometric and distributional properties**

**S2) Simulation studies**

**S3) Missing data**

**S4) Plot of centrality indices for the autism group**

**S5) Plot of bootstrapped confidence intervals around the edge-weights for the autism group**

**S6) Plot of centrality indices for the comparison group**

**S7) Plot of bootstrapped confidence intervals around the edge-weights for the comparison group**

**S8) Plot of centrality indices for the two autism subgroups: “Feelings of High Grip” and “Feelings of Low Grip”**

**S9) Plot of bootstrapped confidence intervals around the edge-weights for the autism subgroups: “Feelings of High Grip” and “Feelings of Low Grip”**

**S10) Results of Network Comparison Tests (NCT)**

**S11) References**

**S1) Measures: Psychometric and distributional properties**

sTable 1.
Included measures, use and psychometric qualities.

| **Measure** | **Use** | **Psychometric qualities** |
| --- | --- | --- |
| Autism characteristics | Sum score, 5 subscales, 2 subscales | Baron-Cohen et al., 2001  Hoekstra et al., 2008 Radhoe et al., 2023 |
| Sensory sensitivity | Sum score | Lever & Geurts, 2013 Radhoe et al., 2023 |
| Educational level | Dutch Verhage scale (1 to 7) | - |
| Sense of mastery | Sum score | Penninx et al., 1997 Peterson, 1999 Radhoe et al., 2023 |
| Worries | Sum score | Van Der Veen et al., 2014  Radhoe et al., 2023 |
| Physical activity | Sum score, score per physical activity domain | Craig et al., 2003 |
| Negative life events | Sum score | Brugha & Cragg, 1990 |
| Emotional support | Sum score, 4 subscales | Hanssen et al., 2019  Radhoe et al., 2023 |
| Positive and negative affect | 2 subscales | Watson et al., 1988  Radhoe et al., 2023 |
| Cognitive failures | Sum score, 4 subscales | Bridger et al., 2013 |
| Psychological difficulties | Sum score, 9 subscales | Smits et al., 2015 |
| Quality of life | Separate items, 4 subscales | The WHOQOL Group, 1998 |
| Physical illnesses | Sum score, separate health conditions | Central Bureau of Statistics (CBS), 1989 |

**
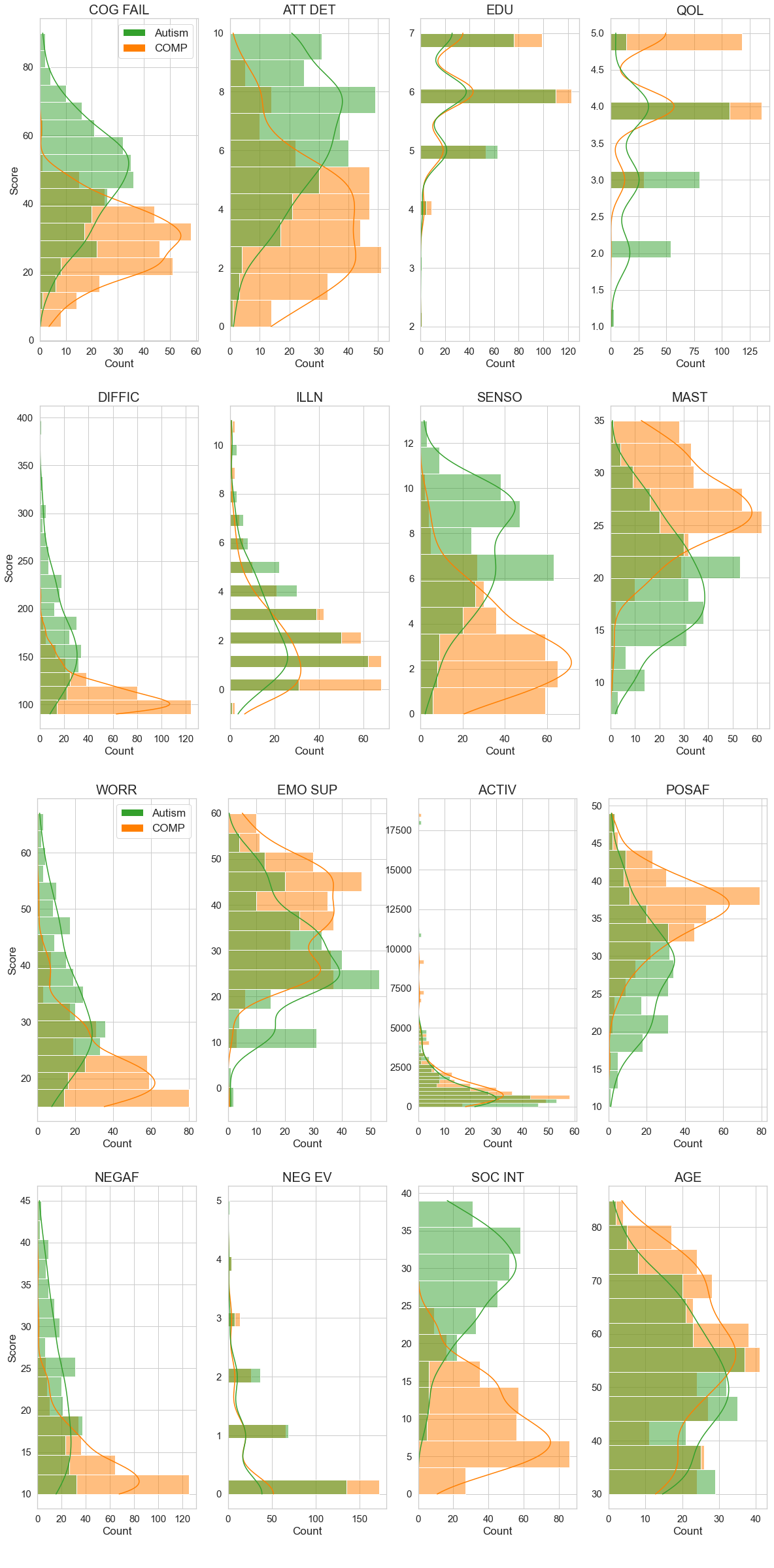
***sFigure 1. Distribution of scores for the autism and comparison groups on the network variables.
Note.* DIFFIC = psychological difficulties, NEGAF = negative affect, WORR = level of worries, QOL = quality of life, SENSO = sensory sensitivity, ATT DET = attention to detail, COG FAIL = cognitive difficulties, EMO SUP = emotional support, SOC INT = social interaction difficulties, ACTIV = physical activity, MAST = mastery, POSAF = positive affect, ILLN = physical illnesses, EDU = education, NEG EV = negative life events.

**S2) Simulation studies**

**Simulations 1 & 2: Required sample size to estimate one network**

For our network study, we aimed to include 16 preselected variables. We performed a simulation study to determine whether we could estimate the networks with sufficient sensitivity and specificity given a sample size varying between 80 and 160 adults. We also varied the percentage of missing data from 0 to 15% to see whether we could estimate the networks in spite of the missing data in our sample.

First, we generated a true network consisting of 16 nodes that we could use to simulate data under. We then simulated a corresponding dataset and estimated a network based on this data. We used the “CompareNetworks” function of the “bootnet” package to determine at what sample size and which amount of missing data, we would end up with a good correspondence between the true network and our estimated network.

Please note that at the time of our preregistration and our simulation studies, we did not know how many subgroups we would identify in our subgrouping study we base these networks on (Radhoe et al., 2023). As we included approximately 400 autistic participants, and we expected to observe two to four subgroups based on the first analyses and literature overview, it was likely that we would have 100 to 200 participants per subgroup. Therefore, we varied the sample size in our simulation study by setting it to 80, 120 and 160 participants. Regarding the percentage of missing data, we simulated 0, 10 and 15% of missingness. We used 50 repetitions in this simulation.


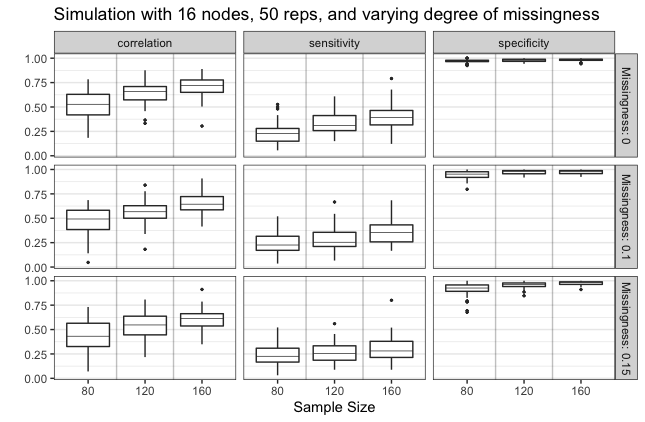


*sFigure 2.* Results of simulations in which the sample size and percentage of missing data are varied to determine the correlation between edge weights, sensitivity and specificity.

Results (see sFigure 2) show that sample sizes of 80, 120, and 160 resulted in a high specificity, but a relatively low sensitivity. Also, 0 to 15% of missing data was associated with similar results across the varying sample sizes.

Therefore, we performed a second simulation, in which we varied the value of alpha to see whether we could improve the sensitivity. We again simulated sample sizes of 80, 120 and 160. Regarding the percentage of missing data, we now chose to simulate 10% of missingness, since it does not greatly affect the results compared to 0% of missingness. Also, in our earlier study in which we identified the subgroups (Radhoe et al., 2023), we performed our community detection analyses with 10% of missing data. We set the alpha-value at 0.05 (which is the default) and 0.25 to see how this would affect the results.

**
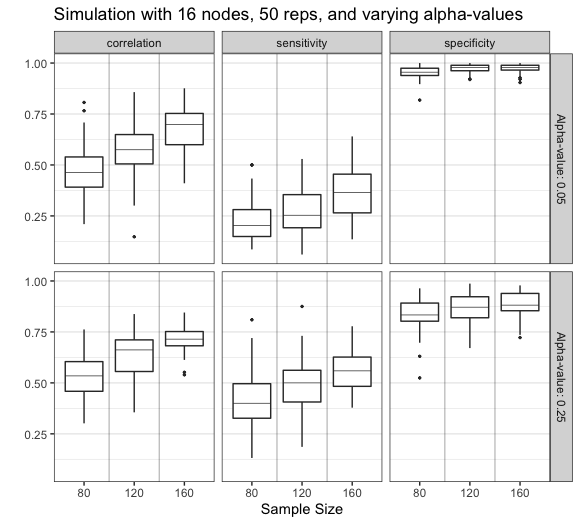
**

*sFigure 3.* Results of simulations in which the sample size and alpha-value are varied to determine the correlation between edge weights, sensitivity and specificity.

The results are presented in sFigure 3. Results show that using *α*=0.25 results in a higher sensitivity while the specificity remains high. Thus, based on these simulations we decided to use a maximum of 10% of missing data and an alpha-value of 0.25.

**Simulation 3: Comparing two distinct networks to assess True Positives**

Besides estimating networks, we also aimed to compare the estimated networks in this study. Therefore, we also performed simulations to determine at which sample size we would be able to detect differences between the networks.

In the third simulation, we generated two distinct true networks and simulated two corresponding datasets. Next, we estimated two networks based on these datasets using EBICglasso. We performed a Network Comparison Test (NCT) to see at what sample size and at which gamma-value, we would detect a significant difference between these two distinct estimated networks. We again used 80, 120 and 160 as sample size. We varied the gamma-value from 0 to 0.5.

We looked at two *p*-values resulting from the NCT:

1. Global strength: the sum of the absolute values of all edges (i.e., the connectivity). This test explores whether the overall level of connectivity is equal across the two networks.
2. Maximum difference in edge weights: The *p-*value resulting from the test concerning the maximum difference in edge weights between the two networks.

sTable 2.

Number of True Positives and False Negatives resulting from 50 repetitions of the Network Comparison Test given varying gamma values.

| Gamma | Outcome NCT | Sample size | | |
| --- | --- | --- | --- | --- |
|  |  | 80 | 120 | 160 |
| 0 | Glstrinv.pval |  |  |  |
|  | - False^1^: *p* >.05 | 21 | 18 | 18 |
|  | - True^2^: *p* <.05 | 29 | 32 | 32 |
|  | Nwinv.pval |  |  |  |
|  | - False^1^:  *p* >.05 | 15 | 6 | 3 |
|  | - True^2^:  *p* <.05 | 35 | 44 | 47 |
| 0.25 | Glstrinv.pval |  |  |  |
|  | - False^1^:  *p* >.05 | 29 | 23 | 18 |
|  | - True^2^:  *p* <.05 | 21 | 27 | 32 |
|  | Nwinv.pval, |  |  |  |
|  | - False^1^:  *p* >.05 | 29 | 22 | 10 |
|  | - True^2^:  *p* <.05 | 21 | 28 | 40 |
| 0.50 | Glstrinv.pval |  |  |  |
|  | - False^1^:  *p* >.05 | 40 | 32 | 25 |
|  | - True^2^: *p* <.05 | 10 | 18 | 25 |
|  | Nwinv.pval, |  |  |  |
|  | - False^1^:  *p* >.05 | 40 | 30 | 20 |
|  | - True^2^:  *p* <.05 | 10 | 20 | 30 |

*Note.* Numbers indicate how frequent, out of 50 repetitions, a certain result was obtained. Glstrinv.pval = difference in global strength. Nwinv.pval = maximum difference in edge weights. ^1^ False indicates “No difference between networks” (i.e., False Negative). ^2^ True indicates “Significant difference between networks” (i.e., True Positive).

Results are presented in sTable 2, and indicate that Gamma=0 can best be used to detect differences between networks belonging to two separate (sub)groups.

For global strength, a sample size of 80 would result in a True Positive rate of 58% (i.e., 29 out of 50 repetitions), which is slightly above chance level. A sample size of 160 would result in a True Positive rate of 64% (i.e., 32 out of 50 repetitions).

For the maximum difference in edge weights, with a sample size of 80, a significant difference (i.e., True Positive) would be detected in 70% of cases (i.e., 35 out of 50 repetitions). A sample size of 160 would result in a True Positive rate of 94% (i.e., 47 out of 50 cases).

**Simulation 4: Comparing two networks to assess False Positives**

In the fourth simulation, we aimed to determine the false positive rate when performing the NCT given various sample sizes. Thus, we first generated one true network with two corresponding datasets. Next, we estimated two networks based on these datasets using EBICglasso. Please note that there is only one underlying true network; therefore, there should not be a significant difference between the networks. We performed a NCT to assess the number of False Positives (i.e., we obtain a significant result even though the underlying network is similar). We again used 80, 120 and 160 as sample size. We varied the gamma-value from 0 to 0.5, and used 50 repetitions. The results are presented in sTable 3.

sTable 3.

Number of False Positives and True Negatives resulting from 50 repetitions of the Network Comparison Test given varying gamma values.

| Gamma | Outcome NCT | Sample size | | |
| --- | --- | --- | --- | --- |
|  |  | 80 | 120 | 160 |
| 0 | Glstrinv.pval: |  |  |  |
|  | - False^1^:  *p* >.05 | 44 | 48 | 48 |
|  | - True^2^:  *p* <.05 | 6 | 2 | 2 |
|  | Nwinv.pval |  |  |  |
|  | - False:  *p* >.05 | 47 | 47 | 48 |
|  | - True:  *p* <.05 | 3 | 3 | 2 |
| 0.25 | Glstrinv.pval |  |  |  |
|  | - False:  *p* >.05 | 42 | 45 | 48 |
|  | - True:  *p* <.05 | 8 | 5 | 2 |
|  | Nwinv.pval |  |  |  |
|  | - False:  *p* >.05 | 46 | 47 | 46 |
|  | - True:  *p* <.05 | 4 | 3 | 4 |
| 0.50 | Glstrinv.pval |  |  |  |
|  | - False:  *p* >.05 | 48 | 49 | 47 |
|  | - True:  *p* <.05 | 2 | 1 | 3 |
|  | Nwinv.pval |  |  |  |
|  | - False:  *p* >.05 | 48 | 48 | 47 |
|  | - True:  *p* <.05 | 2 | 2 | 3 |

*Note.* Numbers indicate how frequent, out of 50 repetitions, a certain result was obtained. Glstrinv.pval = difference in global strength. Nwinv.pval = maximum difference in edge ^1^ False indicates “No difference between networks” (i.e., True Negative). ^2^ True indicates “Significant difference between networks” (i.e., False Positive).

Results indicate that Gamma=0.50 provides the lowest False Positive rate when comparing two networks that do not differ. However, as the difference with Gamma=0 is relatively small, and Gamma=0 resulted in the highest True Positive rate, we proceed with Gamma=0.

For global strength, a sample size of 80 would results in a False Positive rate of 12% (i.e., 6 out of 50 repetitions). A sample size of 160 would result in a False Positive rate of 4% (i.e., 2 out of 50 repetitions).

For the maximum difference in edge weights, a sample size of 80 would result in a False Positive rate of 6% (i.e., 3 out of 50 repetitions), and a sample size of 120 would result in a False Positive rate of 4% (i.e., 2 out of 50 repetitions).

Thus, with a maximum of 10% of missing data and α=0.25 or γ=0, we can compare networks with medium power and an acceptable false discovery rate with as few as 80 participants per subgroup. The R-code for the simulations can be found at <https://osf.io/qbh29>.

**S3) Missing data**

sTable 4.
Percentages of missing data for each of the variables in the comparison and autism groups after imputation.

| **Variable** | **Group** | |
| --- | --- | --- |
|  | Comparison (N=384) | Autism (N=261) |
|  | Percentage of missing values (%) | |
| Cognitive failures | 19.9 | 0.4 |
| Quality of life | 20.2 | 0.8 |
| Psychological difficulties | 20.9 | 0.4 |
| Physical illnesses | 20.4 | 1.1 |
| Education | 20.2 | 1.1 |
| Attention to detail | 20.7 | 0.0 |
| Social interaction | 20.7 | 0.0 |
| Sensory sensitivity | 20.2 | 1.9 |
| Mastery | 20.4 | 0.4 |
| Worries | 20.4 | 0.0 |
| Emotional support | 23.3 | 2.7 |
| Physical activity | 26.6 | 2.3 |
| Positive affect | 25.1 | 0.0 |
| Negative affect | 25.1 | 0.0 |
| Negative life events | 25.8 | 1.1 |

In the comparison group, 97 participants were excluded for having too many missing values after imputation procedures. This subset was significantly younger than the group that was included in the network analysis (M_excluded_=52.2, M_included_=55.7; *t*(382)=2.05, *p*=.041), although there were no differences in sex (*χ*^2^(1)=0.35, *p*=0.552). Of this excluded subset (N=97), there were 59 participants who did not have complete data on any of the included questionnaires, except for age and sex. From the remaining participants (N=38), 16 adults did not complete the second questionnaire booklet (i.e., over half of the questionnaires were missing). In this case, it was chosen not to impute the entire second questionnaire booklet. The remaining participants (N=22) completed the second questionnaire booklet, but had too many missing values across both booklets (i.e., data on at least four out of sixteen questionnaires was missing) to be included in the network analysis.

In the autism group, 3 participants were excluded for having too many missing values after imputation procedures. These participants completed both questionnaire booklets but had too many missing values to be included in the network analysis.

**S4) Plot of centrality indices for the autism group**

**
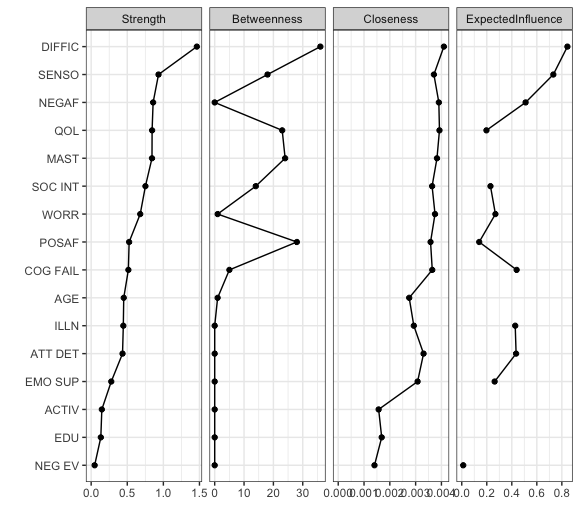
**

*sFigure 4.* Centrality indices for the estimated network of the autism group ordered by node strength.

*Note.* DIFFIC = psychological difficulties, NEGAF = negative affect, WORR = level of worries, QOL = quality of life, SENSO = sensory sensitivity, ATT DET = attention to detail, COG FAIL = cognitive difficulties, EMO SUP = emotional support, SOC INT = social interaction difficulties, ACTIV = physical activity, MAST = mastery, POSAF = positive affect, AGE = biological age, ILLN = physical illnesses, EDU = education, NEG EV = negative life events.

**S5) Plot of bootstrapped confidence intervals around the edge-weights for autism group**

**
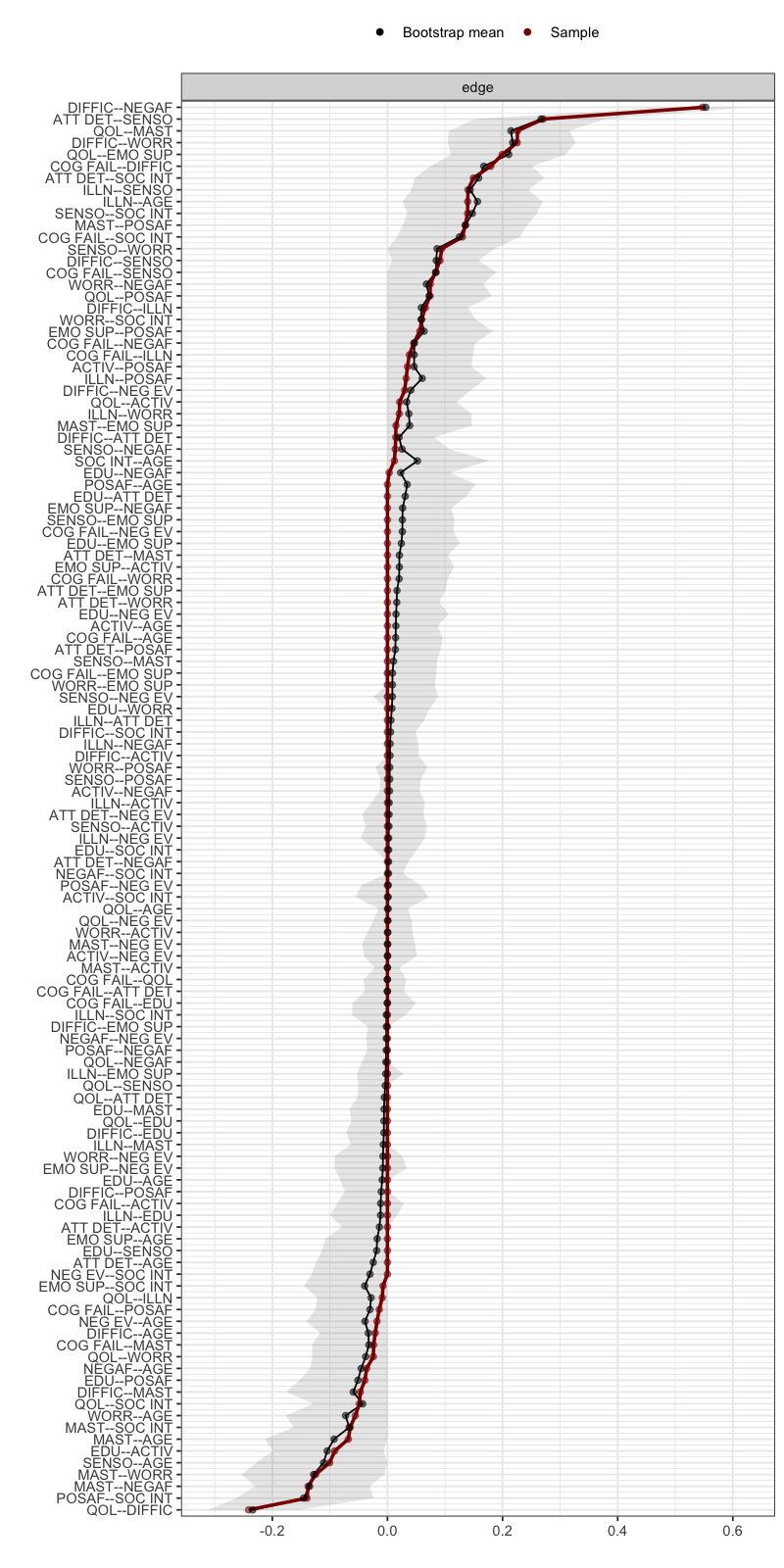
**

*sFigure 5.* Bootstrapped confidence intervals around the estimated edge-weights for the autism group*.*
*Note.* DIFFIC = psychological difficulties, NEGAF = negative affect, WORR = level of worries, QOL = quality of life, SENSO = sensory sensitivity, ATT DET = attention to detail, COG FAIL = cognitive difficulties, EMO SUP = emotional support, SOC INT = social interaction difficulties, ACTIV = physical activity, MAST = mastery, POSAF = positive affect, AGE = biological age, ILLN = physical illnesses, EDU = education, NEG EV = negative life events. The y-axis displays all edges in the network, ordered from the highest edge to the lowest edge weights. The x-axis displays the edge weights. The red dots indicate the value in the sample, the black dots indicate the mean in the bootstrapped samples, and the grey area represents the bootstrapped confidence intervals.

**S6) Plot of centrality indices for the Comparison group**

**
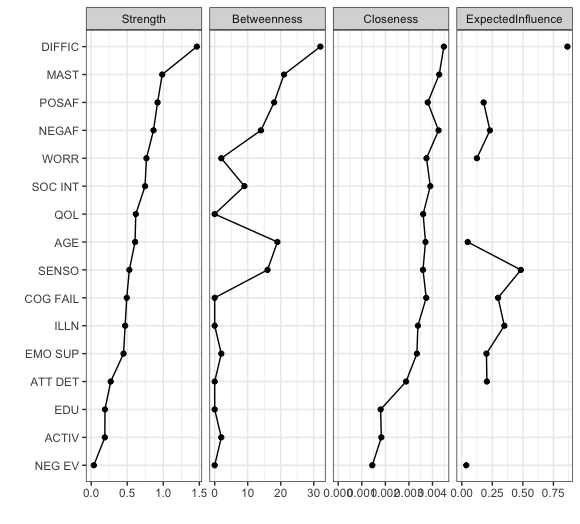
**

*sFigure 6.* Centrality indices for the estimated network of the Comparison group ordered by node strength.

*Note.* DIFFIC = psychological difficulties, NEGAF = negative affect, WORR = level of worries, QOL = quality of life, SENSO = sensory sensitivity, ATT DET = attention to detail, COG FAIL = cognitive difficulties, EMO SUP = emotional support, SOC INT = social interaction difficulties, ACTIV = physical activity, MAST = mastery, POSAF = positive affect, AGE = biological age, ILLN = physical illnesses, EDU = education, NEG EV = negative life events.

**S7) Plot of bootstrapped confidence intervals around the edge-weights for the Comparison group**

**
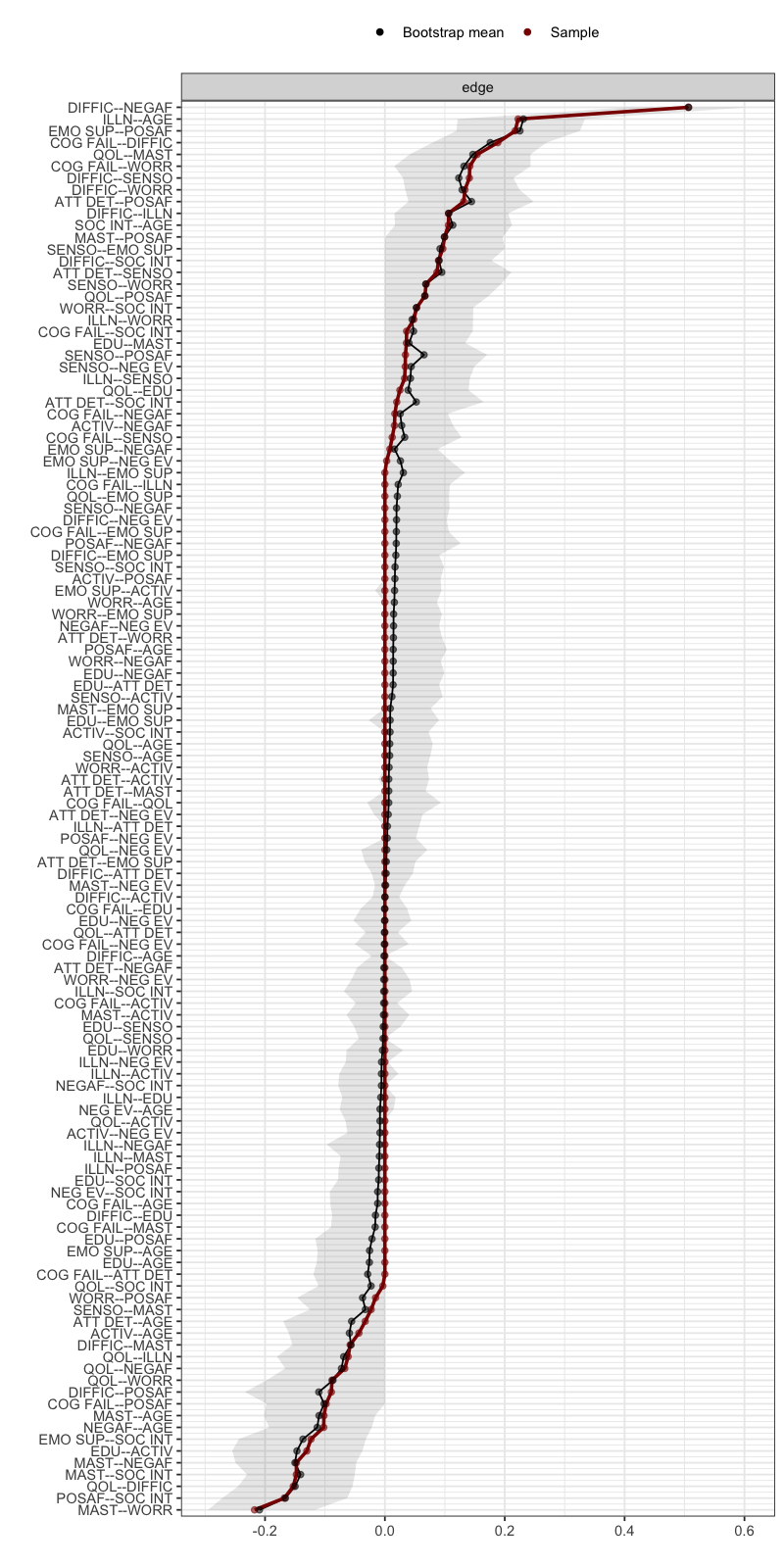
**

*sFigure 7.* Bootstrapped confidence intervals around the estimated edge-weights for the Comparison group.

*Note.* DIFFIC = psychological difficulties, NEGAF = negative affect, WORR = level of worries, QOL = quality of life, SENSO = sensory sensitivity, ATT DET = attention to detail, COG FAIL = cognitive difficulties, EMO SUP = emotional support, SOC INT = social interaction difficulties, ACTIV = physical activity, MAST = mastery, POSAF = positive affect, AGE = biological age, ILLN = physical illnesses, EDU = education, NEG EV = negative life events. The y-axis displays all edges in the network, ordered from the highest edge to the lowest edge weights. The x-axis displays the edge weights. The red dots indicate the value in the sample, the black dots indicate the mean in the bootstrapped samples, and the grey area represents the bootstrapped confidence intervals.

**S8) Plot of centrality indices for the two autism subgroups: “Feelings of High Grip” and “Feelings of Low Grip”**


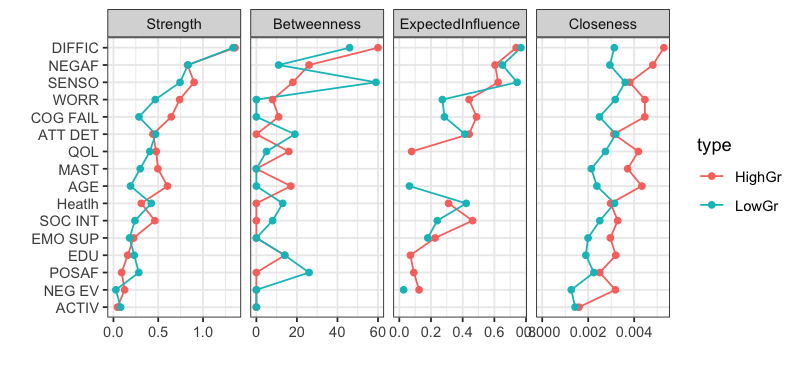


*sFigure 8.* Centrality indices for the estimated networks of the “Feelings of High Grip” and “Feelings of Low Grip” subgroups ordered by node strength.

*Note.* HighGr = Feelings of High Grip, LowGr = Feelings of Low Grip, DIFFIC = psychological difficulties, NEGAF = negative affect, WORR = level of worries, QOL = quality of life, SENSO = sensory sensitivity, ATT DET = attention to detail, COG FAIL = cognitive difficulties, EMO SUP = emotional support, SOC INT = social interaction difficulties, ACTIV = physical activity, MAST = mastery, POSAF = positive affect, AGE = biological age, ILLN = physical illnesses, EDU = education, NEG EV = negative life events.

**S9) Plot of bootstrapped confidence intervals around the edge-weights for the autism subgroups: “Feelings of High Grip” and “Feelings of Low Grip”**


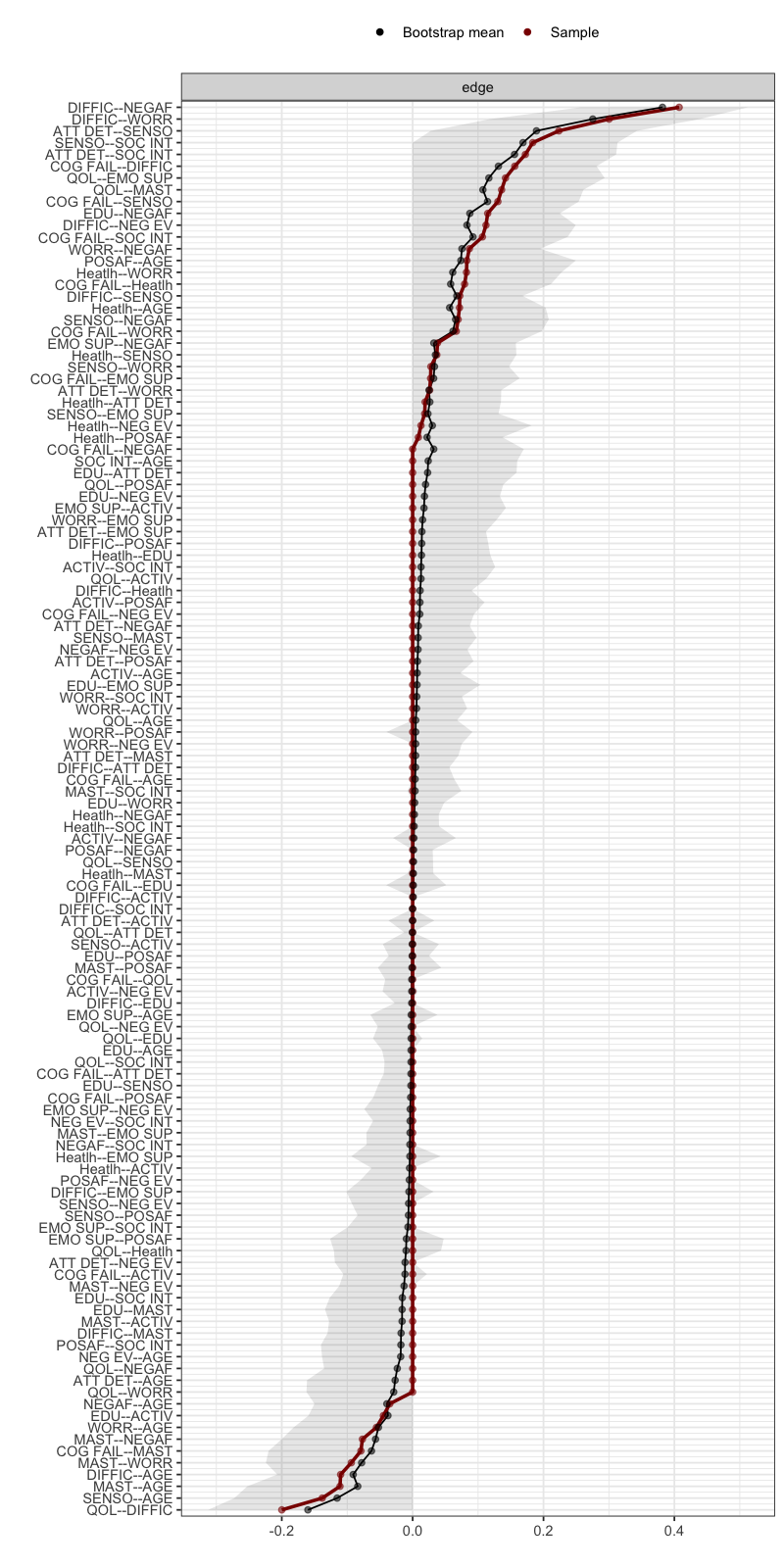


*sFigure 9.* Bootstrapped confidence intervals around the estimated edge-weights for the “Feelings of High Grip” subgroup.

*Note.* DIFFIC = psychological difficulties, NEGAF = negative affect, WORR = level of worries, QOL = quality of life, SENSO = sensory sensitivity, ATT DET = attention to detail, COG FAIL = cognitive difficulties, EMO SUP = emotional support, SOC INT = social interaction difficulties, ACTIV = physical activity, MAST = mastery, POSAF = positive affect, AGE = biological age, ILLN = physical illnesses, EDU = education, NEG EV = negative life events. The y-axis displays all edges in the network, ordered from the highest edge to the lowest edge weights. The x-axis displays the edge weights. The red dots indicate the value in the sample, the black dots indicate the mean in the bootstrapped samples, and the grey area represents the bootstrapped confidence intervals.


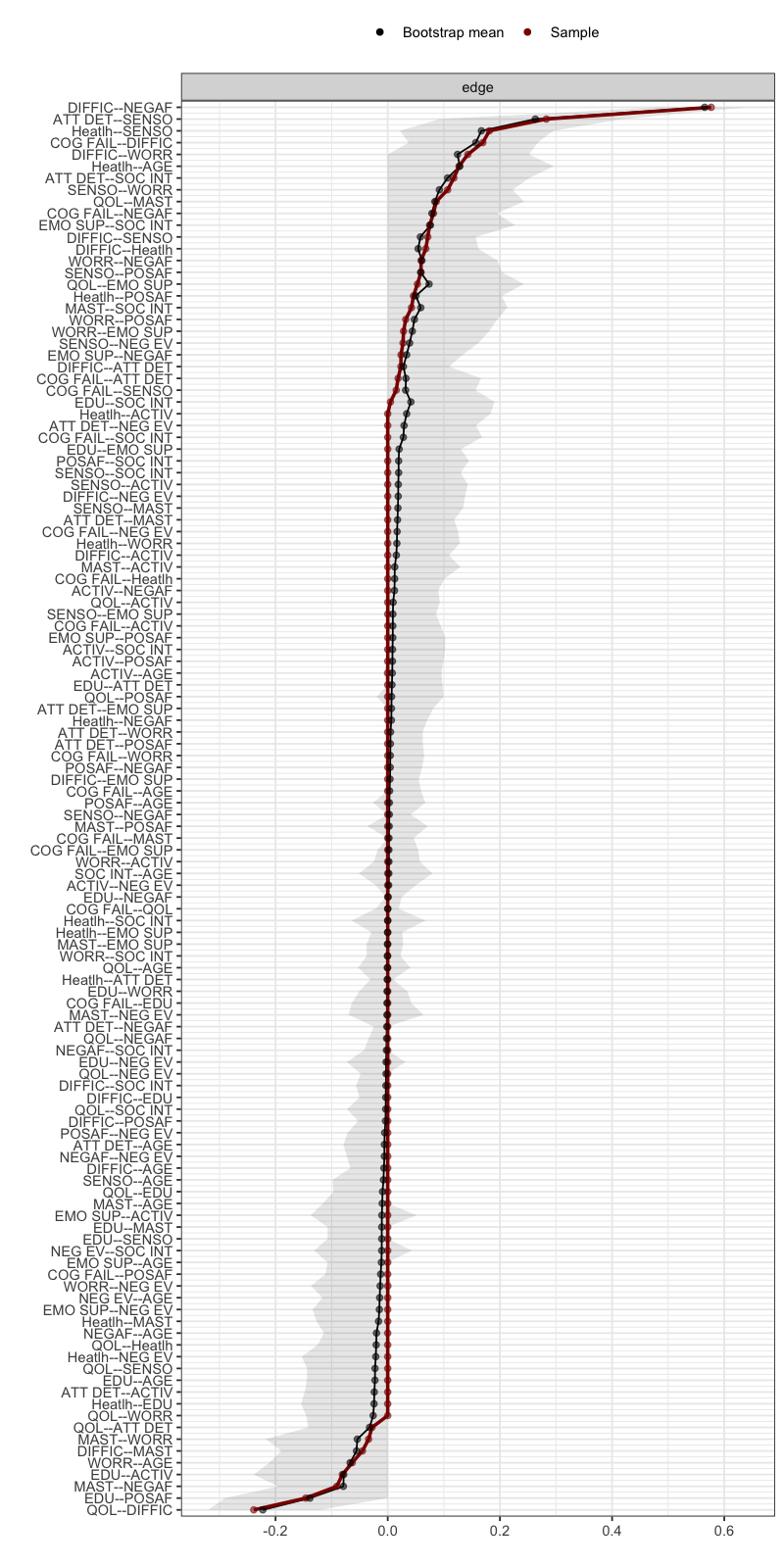


*sFigure 10.* Bootstrapped confidence intervals around the estimated edge-weights for the “Feelings of Low Grip” subgroup.

*Note.* The y-axis displays all edges in the network, ordered from the highest edge to the lowest edge weights. The x-axis displays the edge weights. The red dots indicate the value in the sample, the black dots indicate the mean in the bootstrapped samples, and the grey area represents the bootstrapped confidence intervals.

**S10) Results of Network Comparison Tests (NCT)**

sTable 5.

P-values per edge from the permutation test concerning differences in edge weights based on comparing the autism group vs. comparison group.

| **Variable 1** | **Variable 2** | **P-value** |
| --- | --- | --- |
| ATT DET | SENSO | 0.01 |
| QOL | EMO SUP | 0.02 |
| DIFFIC | POSAF | 0.04 |
| ATT DET | POSAF | 0.01 |
| SENSO | POSAF | <0.01 |
| EMO SUP | POSAF | <0.01 |
| QOL | NEGAF | 0.05 |
| DIFFIC | SOC INT | <0.01 |
| ATT DET | SOC INT | 0.03 |
| EMO SUP | SOC INT | 0.05 |
| SOC INT | AGE | 0.02 |

*Note.* DIFFIC = psychological difficulties, NEGAF = negative affect, QOL = quality of life, SENSO = sensory sensitivity, ATT DET = attention to detail, EMO SUP = emotional support, SOC INT = social interaction difficulties, POSAF = positive affect, AGE = biological age.

sTable 6.

P-values per edge from the permutation test concerning differences in edge weights based on comparing the autism subgroups (“Feelings of High Grip” vs. “Feelings of Low Grip”).

| **Variable 1** | **Variable 2** | **P-value** |
| --- | --- | --- |
| DIFFIC | WORR | 0.02 |
| EDU | POSAF | 0.04 |
| SENSO | POSAF | 0.02 |
| DIFFIC | NEGAF | 0.01 |

*Note.* DIFFIC = psychological difficulties, WORR = worries/fears, EDU = education, NEGAF = negative affect, POSAF = positive affect, SENSO = sensory sensitivity.

**S11) References**

Baron-Cohen, S., Wheelwright, S., Skinner, R., Martin, J., & Clubley, E. (2001). The Autism-Spectrum Quotient (AQ): Evidence from Asperger syndrome/high-functioning autism, males and females, scientists and mathematicians. *Journal of Autism and Developmental Disorders*, *31*(1), 5–17. https://doi.org/10.1023/A:1005653411471

Bridger, R. S., Johnsen Svein  Åge Kjøs, & Brasher, K. (2013). Psychometric properties of the Cognitive Failures Questionnaire. *Ergonomics*, *56*(10), 1515–1524. https://doi.org/https://doi.org/10.1080/00140139.2013.821172

Brugha, T., & Cragg, D. (1990). The List of Threatening Experiences: the reliability and validity of a brief life events questionnaire. *Acta Psychiatrica Scandinavica*, *82*(1), 77–81. https://doi.org/https://doi.org/10.1111/j.1600-0447.1990.tb01360.x

Central Bureau of Statistics (CBS). (1989). *Health Interview Questionnaire*. Central Bureau of Statistics.

Craig, C. L., Marshall, A. L., Sjöström, M., Bauman, A. E., Booth, M. L., Ainsworth, B. E., Pratt, M., Ekelund, U., Yngve, A., Sallis, J. F., & Oja, P. (2003). International physical activity questionnaire: 12-Country reliability and validity. *Medicine and Science in Sports and Exercise*, *35*(8), 1381–1395. https://doi.org/10.1249/01.MSS.0000078924.61453.FB

Hanssen, D. J. C., Rabeling-Keus, I. M., Lucassen, P. L. B. J., Naarding, P., van den Brink, R. H. S., Comijs, H. C., Penninx, B. W. J. H., & Oude Voshaar, R. C. (2019). Measuring social support in psychiatric patients and controls: Validation and reliability of the shortened Close Persons Questionnaire. *Journal of Psychiatric Research*, *116*, 118–125. https://doi.org/10.1016/j.jpsychires.2019.06.006

Hoekstra, R. A., Bartels, M., Cath, D. C., & Boomsma, D. I. (2008). Factor structure, reliability and criterion validity of the autism-spectrum quotient (AQ): A study in Dutch population and patient groups. *Journal of Autism and Developmental Disorders*, *38*(8), 1555–1566. https://doi.org/10.1007/s10803-008-0538-x

Lever, A. G., & Geurts, H. M. (2013). Een nieuw instrument voor sensorische gevoeligheid. *Wetenschappelijk Tijdschrift Autisme*, *2*, 68–73.

Penninx, B. W. J. H., Kriegsman, D. M. W., Boeke, A. J. P., Van Eijk, J. Th. M., Van Tilburg, T., & Deeg, D. J. H. (1997). Effects of social support and personal coping resources on mortality in older age: The longitudinal aging study Amsterdam. *American Journal of Epidemiology*, *146*(6), 510–519. https://doi.org/10.1093/oxfordjournals.aje.a009305

Peterson, C. L. (1999). *Stress at work. A sociological perspective*. CRC Press.

Radhoe, T. A., Agelink van Rentergem, J. A., Torenvliet, C., Groenman, A. P., van der Putten, W. J., & Geurts, H. M. (2023). Finding Similarities in Differences Between Autistic Adults: Two Replicated Subgroups. *Journal of Autism and Developmental Disorders*. https://doi.org/10.1007/s10803-023-06042-2

Smits, I. A. M., Timmerman, M. E., Barelds, D. P. H., & Meijer, R. R. (2015). The Dutch Symptom Checklist-90-Revised: Is the use of the subscales justified? *European Journal of Psychological Assessment*, *31*(4), 263–271. https://doi.org/10.1027/1015-5759/a000233

THE WHOQOL GROUP. (1998). Development of the World Health Organization WHOQOL-BREF Quality of Life Assessment. *Psychological Medicine*, *28*(3), 551–558. https://doi.org/10.1017/S0033291798006667

Van Der Veen, D. C., Comijs, H. C., Van Zelst, W. H., Schoevers, R. A., & Oude Voshaar, R. C. (2014). Defining anxious depression in later life: A scaring heterogeneity in results. *American Journal of Geriatric Psychiatry*, *22*(11), 1375–1378. https://doi.org/10.1016/j.jagp.2014.02.012

Watson, D., Clark, L. A., & Tellegen, A. (1988). Development and validation of brief measures of positive and negative Affect: The PANAS scales. *Journal of Personality and Social Psychology*, *54*(6), 1063–1070. https://doi.org/10.1037/0022-3514.54.6.1063
